# Activation of p53 by scaffold-stabilised expression of Mdm2-binding peptides: visualisation of reporter gene induction at the single-cell level

**DOI:** 10.1038/sj.bjc.6602143

**Published:** 2004-09-21

**Authors:** G B Karlsson, A Jensen, L F Stevenson, Y L Woods, D P Lane, M S Sørensen

**Affiliations:** 1 Pharmexa A/S, Kogle Allé 6, DK-2970 Hørsholm, Denmark; 2 Cancer Research UK Cell Transformation Group, University of Dundee, UK

**Keywords:** peptides, scaffold protein, retroviral vector, p53 activity

## Abstract

Small peptides that perturb intracellular signalling pathways are useful tools in the identification and validation of new drug targets. To facilitate the analysis of biologically active peptides, we have developed retroviral vectors expressing an intracellular scaffold protein that significantly enhances the stability of small peptides in mammalian cells. This approach was chosen because retroviral transduction results in efficient and controlled delivery of the gene encoding the effector peptide, while the scaffold protein not only stabilises the peptide but also facilitates the analysis and potential isolation of the target protein. Here, we have adapted a p53-responsive reporter assay to flow cytometry to demonstrate the versatility of this approach by using peptides with known Mdm2-binding activities inserted into a stable scaffold protein that is suitable for intracellular expression in multiple compartments of mammalian cells. This strategy should be generally applicable to the study of small biologically active peptides in diverse functional assays.

The use of cellular assays to analyse the biological activities of short peptides is a powerful approach for validating peptide modulators of signalling pathways and for mapping functional protein domains (Bottger *et al*, 1996, 1997; Souroujon and Mochly-Rosen, 1998). Characterisation of peptides derived from natural protein sequences, or retrieved from the screening of random peptide libraries, can be used to identify agonists or antagonists of therapeutic relevance. However, the technical limitations associated with analyses of peptides can be significant, both due to the poor half-life of short peptides in the intracellular milieu of mammalian cells and because transfer of most peptides across cellular membranes is inefficient. Small protein scaffolds have been widely used in the screening of random peptide libraries by phage display (Ladner, 1995; Nygren and Uhlen, 1997; Zwick *et al*, 1998). In contrast, there are only few reports on scaffolds suitable for intracellular peptide display in mammalian cells (Bottger *et al*, 1997; Abedi *et al*, 1998; Gururaja *et al*, 2000; Hitoshi *et al*, 2003).

In this study, we have used retroviral vectors to achieve efficient delivery of effector molecules into mammalian cells and we engineered peptides with known p53-inducing activities into a small stable scaffold protein, chymotrypsin inhibitor 2 (CI2), a protease inhibitor naturally present in the seeds of barley (McPhalen and James, 1987). Previous studies have shown that the active loop structure of CI2 can be modified by insertions, deletions and amino-acid substitutions, without the overall stability of the protein being affected (Osmark *et al*, 1993). Moreover, CI2 does not contain any disulphide bonds, thus making it a suitable scaffold protein for expression in any subcellular compartment in mammalian cells. Two p53-activating peptides were used in this study, 12.1 and Arf37. Both peptides bind Mdm2, an E3 ubiquitin ligase responsible for the rapid degradation of p53 observed in unchallenged healthy cells (Honda *et al*, 1997). The 12.1 peptide was identified in a phage display screen for Mdm2-binding peptides (Bottger *et al*, 1996), while the Arf37 peptide is derived from the N-terminal end of the mouse p19^ARF^ protein (Weber *et al*, 2000). Mdm2 is an attractive drug target (Bottger *et al*, 1997; Zheleva *et al*, 2003; Vassilev *et al*, 2004) and these peptides serve to illustrate this. By comparing the activity of 12.1 and Arf37 expressed as free peptides (unconstrained) or presented by CI2 (constrained), we show that scaffold-mediated stabilisation significantly enhances the activity of small peptides. To our knowledge, such direct comparative data have not been reported previously. We propose that scaffold-aided peptide presentation combined with retroviral transduction has broad applications for the validation of biologically active peptides and for the elucidation of intracellular signalling pathways in mammalian cells.

## MATERIALS AND METHODS

### Cells

The T22 reporter cells are stably transfected with a p53-responsive reporter construct, pRGCd-Fos-lacZ, as described elsewhere (Hupp *et al*, 1995; Bottger *et al*, 1997). The T22 cells were cultured in DMEM containing 10% newborn calf serum. The Bosc23 packaging cells, the anti-human NGFR hybridoma (HB-8737) (ATCC) and U2-OS cells were cultured in DMEM containing 10% foetal calf serum.

### Genetic constructs

The ncmΔNGFR bicistronic retroviral vector is derived from the CMVbipep vector (Duch *et al*, 1999), in which the neomycin resistance gene present downstream of the internal ribosomal entry site (IRES) element has been exchanged for a truncated form of the human nerve growth factor receptor, ΔNGFR (Mavilio *et al*, 1994). For structurally constrained presentation of peptides, a 64 amino-acid version of CI2 was employed as a scaffold protein (McPhalen and James, 1987). CI2 was inserted into ncmΔNGFR upstream of the IRES element to generate ncmΔNGFR-CI2 and ncmΔNGFR-nuclear localisation signal (NLS)-CI2; the latter contains the NLS of SV40 T antigen placed N-terminally in frame with CI2. Peptides were inserted into CI2 by replacing the sequence corresponding to the six natural amino acids in the active site loop of CI2 (IVTMEY) with the sequence encoding the relevant heterologous peptide sequences (12.1, 12.1-Ala, Arf15, Arf37). Sequences encoding unconstrained peptides were inserted directly upstream of the IRES element in ncmΔNGFR. As a positive control in the p53 reporter assay, the gene encoding full-length human p14^ARF^ was inserted into the ncmΔNGFR to generate ncmΔNGFR-huARF.

### Cytological techniques

Cell populations were stained for the ΔNGFR transduction marker using the anti-NGFR mAb, followed by an RPE conjugated secondary antibody (goat anti-mouse F(ab)2-RPE, DAKO). p53-dependent *β*-gal activity induced by the different effector peptides was measured by loading the cells with a substrate for *β*-gal, fluorescein di-*β*-D-galactopyranoside (FDG, SIGMA), which is cleaved in *β*-gal-positive cells to yield fluorescein (Nolan *et al*, 1988). Cell cycle analysis was performed by gentle ethanol fixation, RNAse treatment and propidium iodide (PI) staining (50 *μ*g ml^−1^ PI in PBS for 1 h at room temperature (RT)). Data were acquired using a FACS Vantage flow cytometer.

### Transfections

U2-OS cells were plated in 10 cm dishes and grown to 90% confluency. Cells were transfected using Lipofectamine™ 2000 (Invitrogen) with 5–20 *μ*g plasmid DNA per dish and incubated for an additional 24 h. MG132 (10 *μ*M final concentration) was added for the final 4 h of incubation.

### Cell lysis and immunoprecipitations

Cells from each 10 cm dish were lysed on ice in 700 *μ*l NP-40 lysis buffer (50 mM Hepes (pH 7.5), 100 mM NaCl, 5 mM EDTA, 0.5% NP-40) containing protease inhibitor cocktail Complete™ (Roche Diagnostics). The protein concentration of lysates was determined using a BCA™ Protein Assay Kit (Pierce) and lysate volumes adjusted to give equivalent total protein concentrations. In all, 650 *μ*l of each adjusted lysate (in NP-40 lysis buffer+1 mM DTT) was mixed with 20 *μ*l protein G Sepharose coupled to the 4B2 antibody (Chen *et al*, 1993) and incubated, with constant rotation, for 1 h at 4°C. Immunoprecipitates were washed 4 × in NP-40 lysis buffer+1 mM DTT and eluted in 60 *μ*l NP-40 lysis buffer+100 mM DTT.

### Gel electrophoresis and Western blotting

Samples were run on 4–12% Bis–Tris gels in MES running buffer (Novex) and transferred to nitrocellulose overnight at 15 mA. Membranes were blocked for 2 h in PBS+0.1% Tween (PBST)+5% nonfat milk. Primary antibodies 4B2, DO-1 (Bartek *et al*, 1993; Stephen *et al*, 1995), *α*-CI2A, or *α*-Arf (both polyclonal rabbit antisera) were incubated for 1 h at RT. Blots were washed 4 × with PBST and incubated with *α*-mouse-HRP or *α*-rabbit-HRP (Jackson) for 45 min at RT and then washed 4 × with PBST and 1 × with PBS. The signal was generated with ECL (Amersham Biosciences) or Dura (Pierce) substrate and exposed to a film.

### Immunofluorescence

U2-OS cells were seeded at a density of 2.5 × 10^5^ cells ml^−1^ in two-well permanox chamber slides and transiently transfected with plasmid DNA encoding human Mdm2, the CI2 scaffold proteins, or full-length human Arf, using Lipofectamine as described above. At 24 h post-transfection, the cells were fixed and permeabilised for 8 min in ice-cold methanol/acetone 50%/50% (v v^−1^), blocked for 40 min at RT with 0.1% (w v^−1^) BSA in PBS and incubated for 1 h at RT with primary antibodies diluted in DMEM, 10% (v v^−1^) FCS. (*α*-CI2 polyclonal antibody and 4B2 ascites were diluted 1 : 100 each, *α*-Arf polyclonal antibody was diluted 1 : 1000.) Following incubation with secondary antibodies (30 min, RT), FITC-conjugated anti-mouse IgG (1 : 80) and TRITC-conjugated anti-rabbit IgG (1 : 400), the cells were washed extensively in PBS, fixed in Dapi, mounted with Hydromount and visualised by microscopy on a Nikon Eclipse E600 microscope.

## RESULTS

### Design of the retroviral vector

**Figure 1:**
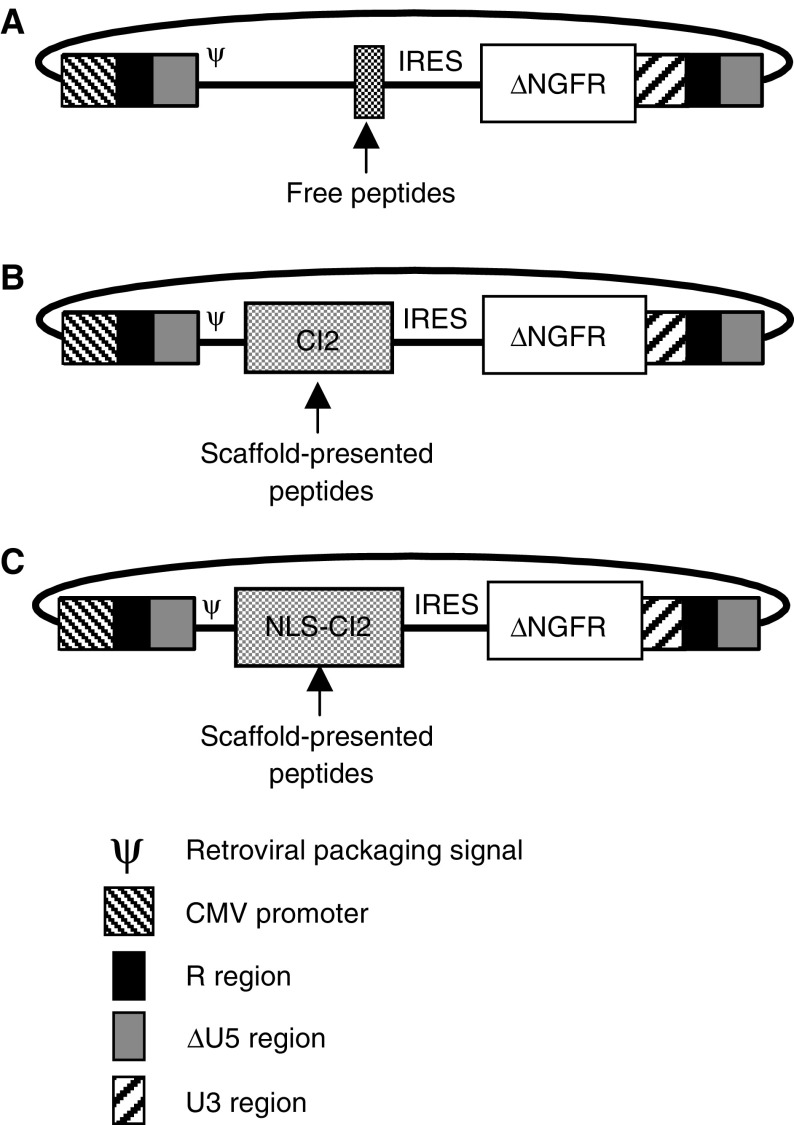
Design of retroviral vectors used for peptide expression. All vectors contain a chimeric 5′ LTR. Transcription in transfected cells is driven by the cytomegalovirus (CMV) promoter and in transduced cells by the retroviral LTR. An IRES element facilitates CAP-independent translation of the ΔNGFR transduction marker. The ncmΔNGFR vector (**A**) was used for expression of unconstrained peptides and full-length proteins, while the ncmΔNGFR-CI2 (**B**) and the ncmΔNGFR-NLS-CI2 (**C**) vectors were used for expression of constrained peptides.

Bicistronic retroviral vectors based on the murine Akv retrovirus were used for efficient transduction of mammalian cells (Duch *et al*, 1999). The ncmΔNGFR vector was used for the expression of unconstrained peptides and full-length control proteins (Figure 1A

), while structurally constrained peptides were expressed from the ncmΔNGFR-CI2 (Figure 1B) or from ncmΔNGFR-NLS-CI2 (Figure 1C). Long-term overexpression of CI2 in mammalian cells did not result in cellular toxicity (A Jensen *et al*, unpublished).

### CI2 presentation of an Mdm2-binding peptide: visualisation of reporter gene induction at the single-cell level

**Figure 2:**
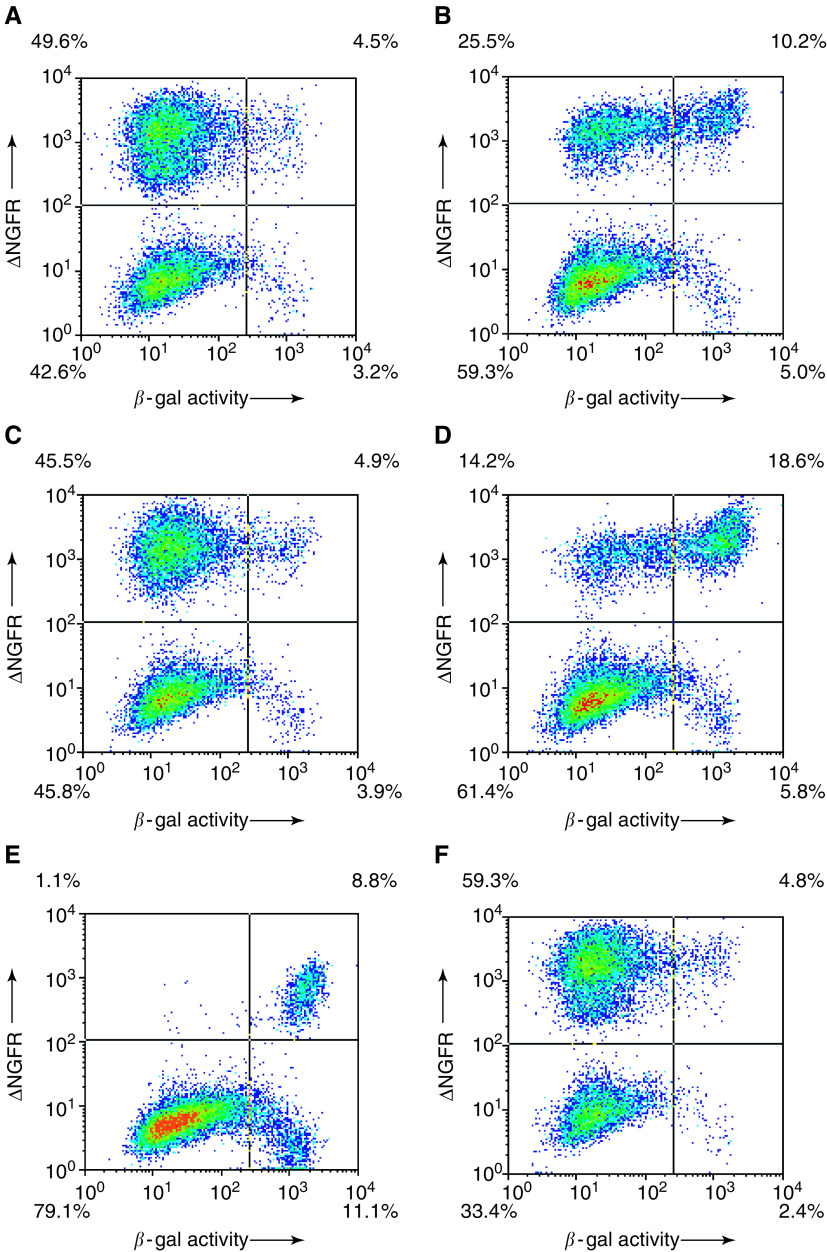
Visualisation of p53 activity at the single-cell level. T22 cells were analysed 68 h after transduction by staining for ΔNGFR (*y*-axis) and by assaying for *β*-gal activity (*x*-axis). Density plots from representative experiments for each vector are shown: (**A**) ncmΔNGFR-CI2-12.1-Ala; (**B**) ncmΔNGFR-CI2-12.1; (**C**) ncmΔNGFR-NLS-CI2-12.1-Ala; (**D**) ncmΔNGFR-NLS-CI2-12.1; (**E**) ncmΔNGFR-huArf and (**F**) and ncmΔNGFR-CI2.

Examples of the data obtained in the flow cytometry-based p53 reporter assay are shown as density plots in Figure 2

. A marked increase in p53-driven *β*-gal activity was observed in ΔNGFR-positive cultures expressing the CI2-presented 12.1 peptide (Figure 2B and D), compared to cultures expressing the CI2-presented 12.1-Ala control peptide (Figure 2A and C). The addition of an NLS to the CI2-12.1 protein further increased the number of *β*-gal-positive cells in the culture (Figure 2B and D). When the expression levels of CI2-12.1 and CI2-12.1-Ala were analysed by Western blot, the two proteins were expressed to equal levels (data not shown). Thus, the difference in reporter gene expression can be ascribed to a difference in biological activity between these proteins. Further, all cells transduced with ncmΔNGFR-huARF were positive for p53-driven *β*-gal activity (Figure 2E) and expression of p53-activating molecules resulted in a lower overall proportion of transduced cells at 68 h after transduction (Figure 2B, D and E), indicating that the growth rate of cells transduced with p53-activating molecules was slowed. This was particularly pronounced for cultures transduced with ncmΔNGFR-huARF, in which less than 10% of the total population was NGFR positive at this time point. These results did not reflect lower transduction efficiencies of the three cultures expressing p53-inducing molecules, since analyses performed at an earlier time point after transduction showed similar proportions of NGFR-positive cells in all cultures (not shown).

### Activity of unconstrained and scaffold-constrained Mdm2-binding peptides

**Figure 3:**
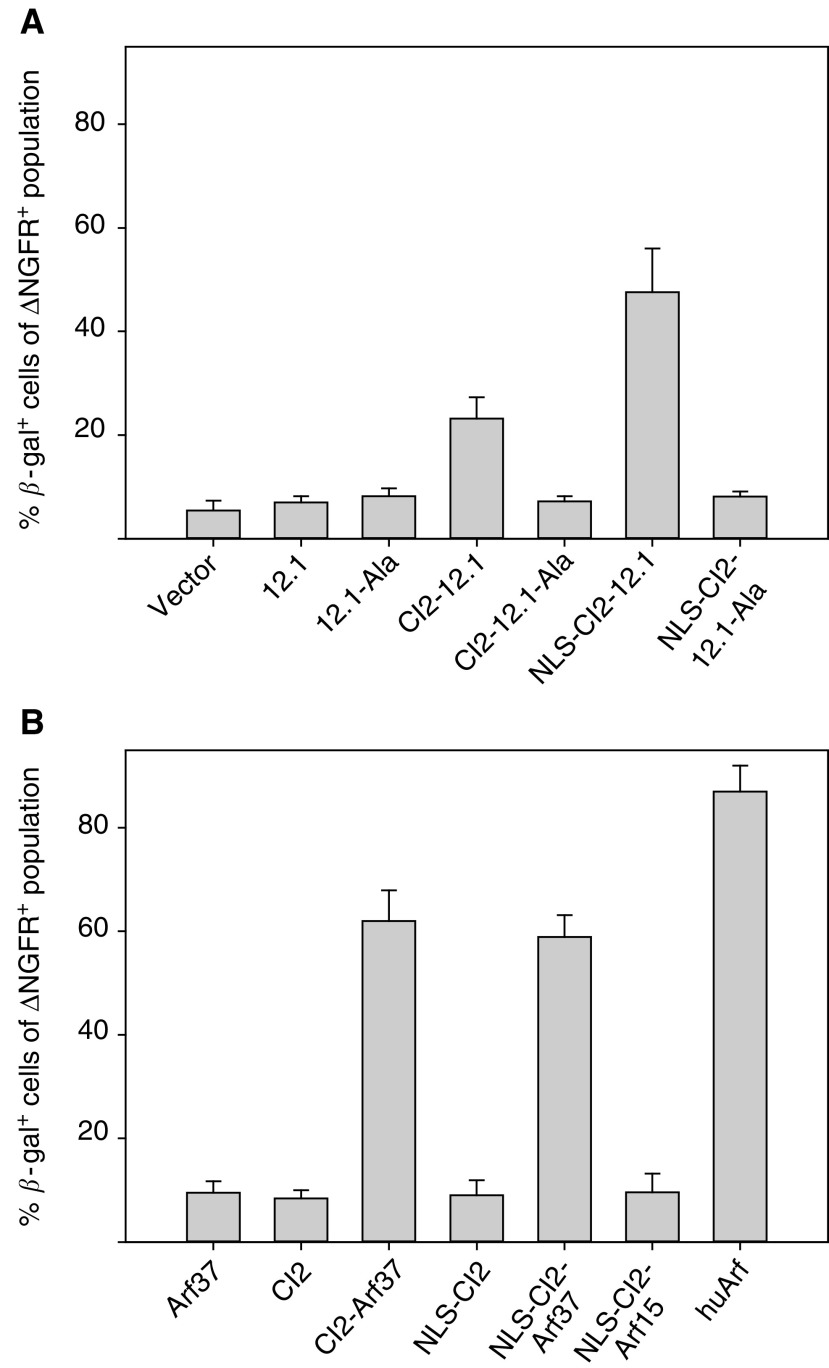
The 12.1 and the Arf37 peptides are stabilised by the CI2 scaffold. T22 reporter cells were transduced with a panel of retroviral vectors expressing the 12.1 peptide and appropriate controls: (**A**) ncmΔNGFR, ncmΔNGFR-12.1, ncmΔNGFR-12.1-Ala, ncmΔNGFR-CI2-12.1, ncmΔNGFR-CI2-12.1-Ala, ncmΔNGFR-NLS-CI2-12.1 and ncmΔNGFR-NLS-CI2-12.1-Ala, (**B**) ncmΔNGFR-Arf37, ncmΔNGFR-CI2, ncmΔNGFR-CI2-Arf37, ncmΔNGFR-NLS-CI2, ncmΔNGFR-NLS-CI2-Arf37, ncmΔNGFR-NLS-CI2-Arf15 and ncmΔNGFR-huArf. Samples were analysed 68 h after transduction by staining the cells for ΔNGFR and assaying for *β*-gal activity. Data are plotted as the percentage of double-positive cells (ΔNGFR and *β*-gal) of the total transduced population (ΔNGFR-positive cells). Results from three independent transductions are shown.

Having established that CI2 is a functional intracellular scaffold protein for presentation of the 12.1 peptide, we wished to determine if this was more broadly applicable to other peptides and we therefore inserted a different Mdm2-binding peptide into CI2, namely a 37 amino-acid large peptide from the N-terminus of p19^ARF^, Arf37. In the same experiment, we also assayed the biological activity of the unconstrained 12.1 and Arf37 peptides expressed from the retroviral vector and determined if the addition of a nuclear targeting signal enhanced the activity of the CI2-presented peptides. When the 12.1 peptide was expressed as a free peptide, no activity over background levels was observed, while CI2-constrained 12.1 peptide activated p53 in approximately 25% of the transduced population (Figure 3A

). The addition of stabilising residues to the N- and C-termini of the unconstrained 12mer peptide (MG-12.1-GGPP) did not result in any detectable increase in activity (data not shown). Targeting CI2 to the nucleus increased the proportion of p53-activated cells to approximately 50% of the total transduced population, likely reflecting an increased nuclear concentration of CI2-12.1 (Figure 3A). All 12.1-Ala control constructs showed background levels of activity, confirming the peptide-specific p53 induction.

CI2 presentation also enhanced the activity of the Arf37 peptide (Figure 3B), demonstrating that it can improve the stability of quite sizeable peptides. As for the 12.1 peptide, the addition of stabilising residues flanking the free Arf37 peptide (MG-Arf37-GGPP) did not improve the activity of the unconstrained Arf37 peptide (data not shown). We ascribe the lack of activity in cells transduced with vectors expressing free (unconstrained) 12.1 and Arf37 to poor stability rather than poor expression of the peptides, since previous studies using another functional assay have shown that short peptides are expressed from this position in the vector (Tolstrup *et al*, 2001). In contrast to the 12.1 peptide, targeting the scaffold to the nucleus did not increase the activity of CI2-Arf37, likely because the Arf37 peptide already contains a sequence of basic residues that confer nuclear localisation. A vector expressing CI2 presenting amino acids 1–15 of p19^ARF^ was also included in the assay, ncmΔNGFR-NLS-CI2-Arf15. This 15mer peptide has been shown to bind Mdm2, but to lack other determinants necessary for p53 induction. In agreement with this, we detected no activity over background levels in cultures transduced with ncmΔNGFR-NLS-CI2-Arf15 peptide. Retroviral delivery of full-length human p14^ARF^ resulted in near 100% reporter assay-positive cells in the transduced population, suggesting that this level of p53-inducing activity represents near saturation in the assay.

### Binding and co-localisation of CI2-Arf37 with endogenous Mdm2

**Figure 4:**
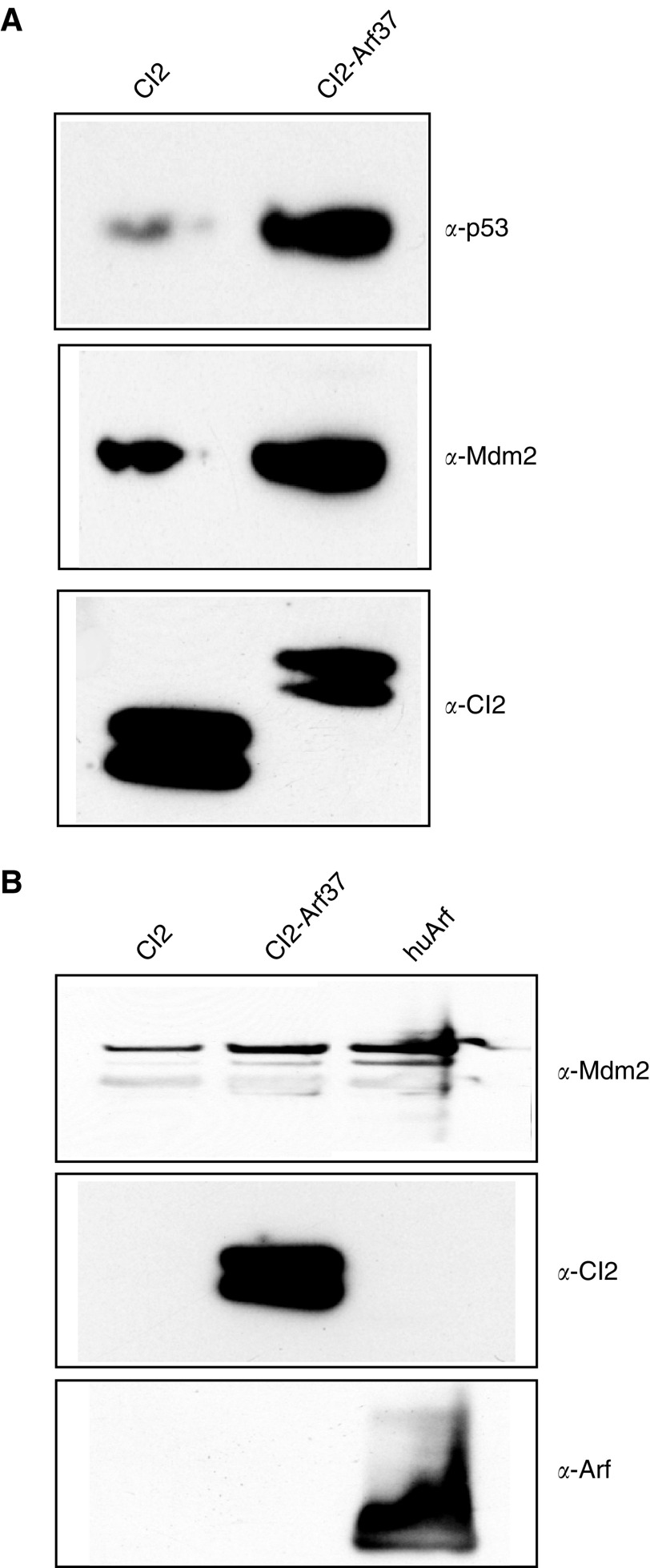
Co-precipitation of CI2-Arf37 and endogenous Mdm2 (**A**). Whole-cell lysates of U2-OS cells transfected with ncmΔNGFR-CI2 or ncmΔNGFR-NLS-CI2-Arf37 were analysed by Western blot using antibodies against p53, Mdm2 or CI2 (**B**). Whole-cell lysates of U2-OS cells transfected with ncmΔNGFR-CI2, ncmΔNGFR-NLS-CI2-Arf37 or human Arf were precipitated with protein G sepharose-coupled *α*-Mdm2. The bound material was eluted and analysed by Western blot using antibodies against Mdm2, CI2 and Arf.

To obtain evidence that the p53-inducing activity of the scaffold-presented peptides resulted from an interaction between the CI2-presented peptides with endogenous Mdm2, co-precipitation experiments were performed. In these experiments CI2-Arf37 was used, since this protein activated p53 potently in the flow cytometry-based assay. First, the expression levels of endogenous Mdm2 and p53 were analysed in CI2- and CI2-Arf37-transfected U2-OS cells (Figure 4A

). In agreement with our previous results from the reporter assay, we found that the level of p53 protein was increased in CI2-Arf37-expressing cells compared to that in cells expressing the empty scaffold protein. Thus, upregulation of p53 was detectable also at the protein level. This experiment also showed that endogenous Mdm2, as well as transfected CI2 and CI2-Arf, were readily detected. We then analysed the ability of endogenous Mdm2 to co-precipitate CI2, CI2-Arf37 and human Arf by running Mdm2-immunoprecipitated material on a gel and performing Western blot analysis with *α*-CI2 and *α*-Mdm2 antibodies (Figure 4B). We observed a clear co-precipitation of CI2-Arf37 and human Arf with Mdm2, while the empty CI2 scaffold did not co-precipitate. These data confirm that CI2-presented Arf37 interacts specifically with endogenous Mdm2.

**Figure 5:**
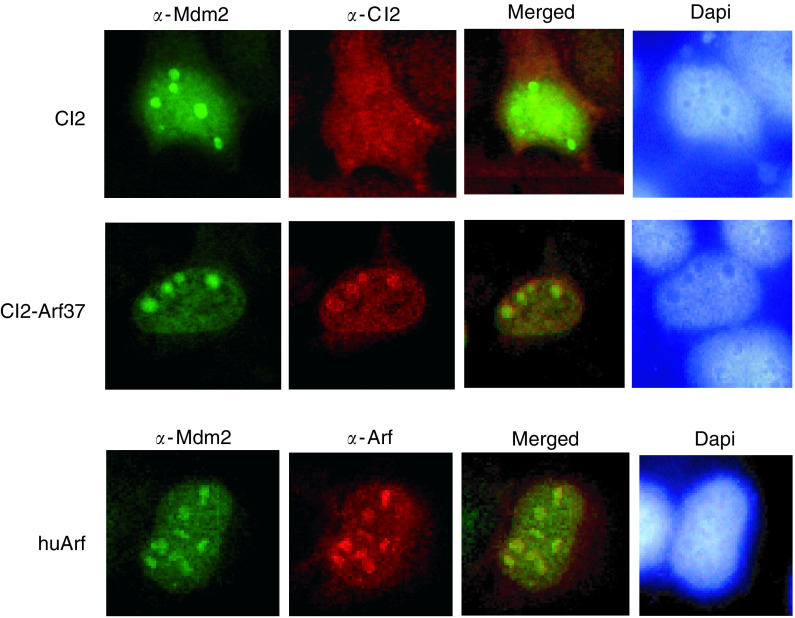
Co-localisation of CI2-Arf37 and Mdm2. U2-OS cells transfected with human Mdm2 and ncmΔNGFR-CI2 (upper row), ncmΔNGFR-NLS-CI2-Arf37 (middle row) or human Arf (lower row) were analysed by immunofluorescence using antibodies against Mdm2 (first column), CI2 or Arf (second column) and by merging the images (third column). The column furthest to the right shows the cell nucleus stained with Dapi.

To demonstrate that an interaction between CI2-Arf37 and Mdm2 occurs also in intact cells, we analysed CI2- and CI2-Arf37-expressing cells by immunofluorescence (Figure 5

). This experiment demonstrates that Mdm2 localises to the nucleus and in particular to the nucleoli. A clear co-localisation with Mdm2 of CI2-Arf37 and human Arf was observed, while the empty CI2 scaffold was distributed in both the cytosol and the nucleus, with no accumulation in the nucleoli. To obtain a clear signal, the data shown in Figure 5 are from cells transiently transfected with human Mdm2; however, a similar co-localisation was also observed when CI2-transfected cells were stained for endogenous Mdm2 (not shown). These results firmly establish the co-localisation of CI2-Arf37 with Mdm2.

### Cell cycle arrest induced by the scaffold-presented 12.1 peptide

**Figure 6:**
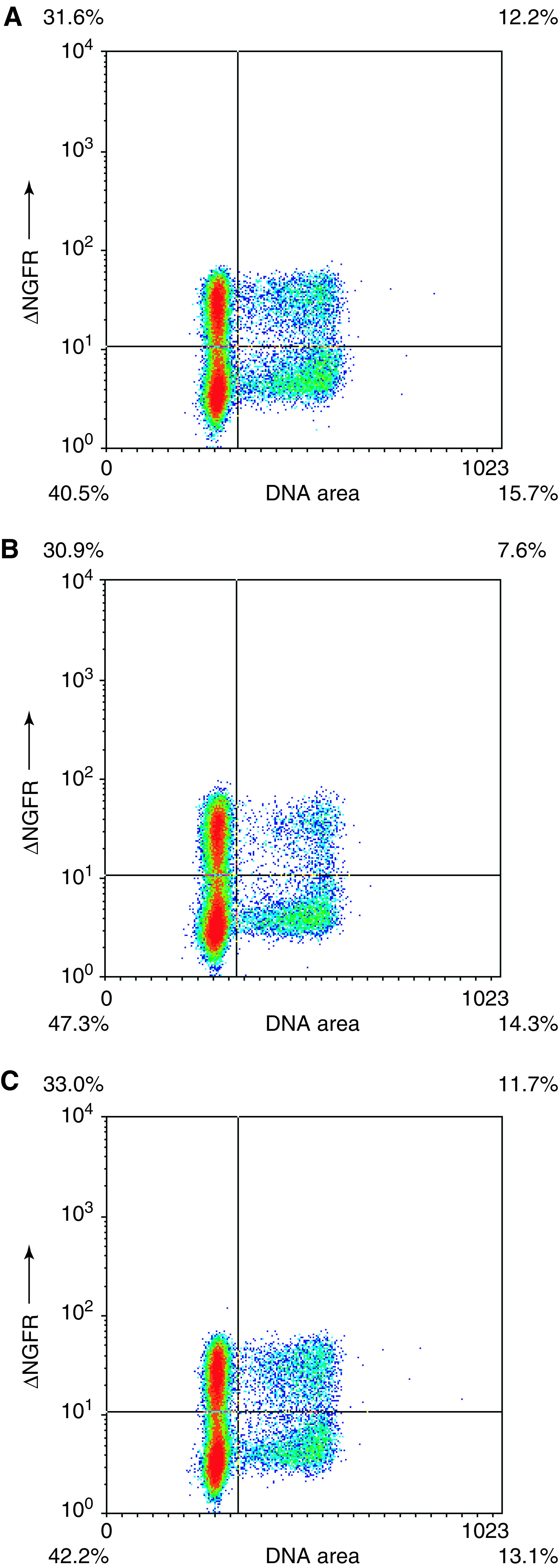
Cells expressing the CI2-presented 12.1 peptide show slowed cell cycle progression. T22 cells transduced with (**A**) ncmΔNGFR; (**B**) ncmΔNGFR-NLS-CI2-12.1 and (**C**) ncmΔNGFR-NLS-CI2-12.1-Ala were stained for ΔNGFR and for DNA content at 68 h after transduction. A representative experiment is shown.

To confirm that biologically relevant activities are measured in the p53 reporter assay, we performed cell cycle analyses by determining the DNA content in T22 cells transduced with ncmΔNGFR-CI2-12.1 and control vectors. Again, we used the ΔNGFR surface marker to distinguish the transduced population from the untransduced in order to take into account the growth disadvantage of cells expressing p53-activating peptides. The results demonstrate that expression of CI2-presented 12.1 reduced the percentage of cells progressing through S phase (Figure 6B

) compared to that in control cultures (Figure 6A and C). Similar results were obtained using CI2-Arf37 (not shown).

## DISCUSSION

In this study, we have combined the use of retroviral transduction methods and scaffold-mediated peptide presentation to circumvent two key limitations associated with the analysis of short peptides inside cells, namely peptide delivery and peptide stability. When using low multiplicities of infection, retroviral transduction results in the delivery of a controlled copy number of the sequence encoding the effector molecule. We found that CI2-stabilised Mdm2-binding peptides induced a potent p53 response, which was readily detected in a flow cytometry-adapted p53-reporter assay. The same peptides did not stimulate a detectable response when expressed as free peptides, likely due to the poor stability of small peptides in mammalian cells, Moreover, we show that CI2-Arf37, a molecule that exhibits potent activity in the flow cytometry-based p53 reporter assay, specifically interacts and co-localises with endogenous Mdm2. These data suggest that CI2 is a versatile scaffold protein, which will prove useful for validation studies and for the presentation of intracellular combinatorial peptide libraries used in functional screens.

Related strategies have been used to identify active peptides from random libraries (Peelle *et al*, 2001; Xu *et al*, 2001; Hitoshi *et al*, 2003) and the vectors presented here could be similarly applied. Finally, by monitoring the total cell population in a flow cytometry-based assay, our data show that cells expressing p53-activating peptides rapidly become outgrown by the untransduced cells in the culture. These results underscore the importance of performing analysis at the single-cell level when studying peptides with growth-inhibitory properties, since cells that express potent effector molecules selectively get depleted from the total cell population.
